# Supplementary material for: Demand for family planning satisfied with modern methods and its associated factors among married women of reproductive age in rural Jordan: A cross-sectional study
Source: PLoS One. 2020 Mar 18;15(3):e0230421. doi: 10.1371/journal.pone.0230421 (PMC7080244; doi:10.1371/journal.pone.0230421)
Supplement: S1 Table — (DOCX) [file pone.0230421.s001.docx]

S1 Table. Number of samples by village

| Health district | Intervention villages | Expected sample number | Actual collected  number | Control  villages | Expected sample number | Actual collected  number |
| --- | --- | --- | --- | --- | --- | --- |
| Irbid | Assa'ara | 85 | 98 | Nattfieh | 123 | 121 |
| Irbid | Mandah | 125 | 148 | Makhraba | 94 | 100 |
| Kura | Kufor Kefia | 63 | 62 | Saydoor | 116 | 118 |
| Al-Aghwar shamaieh | Abu Habil | 92 | 77 | Izmalieh | 91 | 105 |
| Bani Kenanah | Al-khariabeh | 135 | 126 | Esaileh | 76 | 64 |
| Total |  | 500 | 511 |  | 500 | 508 |
| Ground Total | Expected sample number: 1,000  Actually collected number: 1,019 | | | | | |
