# Supplementary material for: Demand for family planning satisfied with modern methods and its associated factors among married women of reproductive age in rural Jordan: A cross-sectional study
Source: PLoS One. 2020 Mar 18;15(3):e0230421. doi: 10.1371/journal.pone.0230421 (PMC7080244; doi:10.1371/journal.pone.0230421)
Supplement: S2 Table — (DOCX) [file pone.0230421.s002.docx]

S2 Table. Participants according to nationality (n=1,019)

|  | n | % |
| --- | --- | --- |
| Jordanian | 973 | 95.5 |
| Syrian | 43 | 4.2 |
| Other | 3 | 0.3 |
