# Supplementary material for: Demand for family planning satisfied with modern methods and its associated factors among married women of reproductive age in rural Jordan: A cross-sectional study
Source: PLoS One. 2020 Mar 18;15(3):e0230421. doi: 10.1371/journal.pone.0230421 (PMC7080244; doi:10.1371/journal.pone.0230421)
Supplement: S3 Table — (DOCX) [file pone.0230421.s003.docx]

S3 Table. Contraceptive methods among women having needs for modern contraception (Multiple answer) (n=762)

|  | n | % |
| --- | --- | --- |
| **All modern methods** | **417** | **54.7** |
| IUD | 247 | 32.4 |
| Injectable | 10 | 1.3 |
| Implants | 4 | 0.5 |
| Pills | 87 | 11.4 |
| Male condom | 47 | 6.2 |
| LAM | 1 | 0.1 |
| Female sterilization | 22 | 2.9 |
| Other modern methods | 1 | 0.1 |
| **All traditional methods** | **202** | **26.5** |
| Rhythm method | 9 | 1.2 |
| Withdrawal | 187 | 24.5 |
| Beast feeding | 6 | 0.8 |
| **No use of any methods** | **143** | **18.8** |
