# Supplementary material for: Demand for family planning satisfied with modern methods and its associated factors among married women of reproductive age in rural Jordan: A cross-sectional study
Source: PLoS One. 2020 Mar 18;15(3):e0230421. doi: 10.1371/journal.pone.0230421 (PMC7080244; doi:10.1371/journal.pone.0230421)
Supplement: S4 Table — (DOCX) [file pone.0230421.s004.docx]

S4 Table. Awareness of the nearest village health centre (n=1,019)

|  | n | % |
| --- | --- | --- |
| Yes | 971 | 95.3 |
| No | 48 | 4.7 |
