# Supplementary material for: Demand for family planning satisfied with modern methods and its associated factors among married women of reproductive age in rural Jordan: A cross-sectional study
Source: PLoS One. 2020 Mar 18;15(3):e0230421. doi: 10.1371/journal.pone.0230421 (PMC7080244; doi:10.1371/journal.pone.0230421)
Supplement: S5 Table — (DOCX) [file pone.0230421.s005.docx]

Table S5. Use of village health centres in the last year (n=971)

|  | n | % |
| --- | --- | --- |
| Yes | 757 | 78.0 |
| No | 214 | 22.0 |
