# Supplementary material for: Demand for family planning satisfied with modern methods and its associated factors among married women of reproductive age in rural Jordan: A cross-sectional study
Source: PLoS One. 2020 Mar 18;15(3):e0230421. doi: 10.1371/journal.pone.0230421 (PMC7080244; doi:10.1371/journal.pone.0230421)
Supplement: S6 Table — (DOCX) [file pone.0230421.s006.docx]

S6 Table. Transportation mean to the nearest village health centre (n=757)

|  | n | % |
| --- | --- | --- |
| On foot | 615 | 81.2 |
| Private car | 125 | 16.5 |
| Taxi | 7 | 0.9 |
| Bus/ minibus | 9 | 1.2 |
| Others | 1 | 0.1 |
| Don't know | 0 | 0.0 |
