# Supplementary material for: Demand for family planning satisfied with modern methods and its associated factors among married women of reproductive age in rural Jordan: A cross-sectional study
Source: PLoS One. 2020 Mar 18;15(3):e0230421. doi: 10.1371/journal.pone.0230421 (PMC7080244; doi:10.1371/journal.pone.0230421)
Supplement: S7 Table — (DOCX) [file pone.0230421.s007.docx]

S7 Table. Time required to reach the nearest village health centre (n=757)

|  | n | % |
| --- | --- | --- |
| <=10 | 611 | 80.7 |
| >10 | 145 | 19.2 |
| Don't know | 1 | 0.1 |
| [Mean] | [8.49] |  |
