# Supplementary material for: Demand for family planning satisfied with modern methods and its associated factors among married women of reproductive age in rural Jordan: A cross-sectional study
Source: PLoS One. 2020 Mar 18;15(3):e0230421. doi: 10.1371/journal.pone.0230421 (PMC7080244; doi:10.1371/journal.pone.0230421)
Supplement: S8 Table — (DOCX) [file pone.0230421.s008.docx]

S8 Table. Services used at village health centre (n=757)

|  | n | % |
| --- | --- | --- |
| Vaccination for children | 284 | 37.5 |
| FP counseling | 8 | 1.1 |
| FP methods | 9 | 1.2 |
| ANC | 12 | 1.6 |
| PNC | 3 | 0.4 |
| Women's disease, | 32 | 4.2 |
| General medical examination | 616 | 81.4 |
| Counseling | 26 | 3.4 |
| Others | 53 | 7.0 |
