# Supplementary material for: Demand for family planning satisfied with modern methods and its associated factors among married women of reproductive age in rural Jordan: A cross-sectional study
Source: PLoS One. 2020 Mar 18;15(3):e0230421. doi: 10.1371/journal.pone.0230421 (PMC7080244; doi:10.1371/journal.pone.0230421)
Supplement: S9 Table — (DOCX) [file pone.0230421.s009.docx]

S9 Table. Reasons for non-use of village health centre (n=214)

|  | n | % |
| --- | --- | --- |
| I don't know what kinds of services they are providing | 4 | 1.9 |
| There are no services I need | 70 | 32.7 |
| There is no medical doctor | 24 | 11.2 |
| Attitude of staff at the VHC is not good | 6 | 2.8 |
| Far from home | 5 | 2.3 |
| Services are limited | 75 | 35.0 |
| Drugs are limited | 42 | 19.6 |
| I am using Comprehensive Center nearby | 53 | 24.8 |
| I am using Primary Health Center nearby | 11 | 5.1 |
| I am using a Hospital | 4 | 1.9 |
| I am using a NGO's Health facility | 29 | 13.6 |
| I am using a private hospital/clinic | 39 | 18.2 |
| Others | 37 | 17.3 |
| Don't know | 2 | 0.9 |
