# Supplementary material for: Demand for family planning satisfied with modern methods and its associated factors among married women of reproductive age in rural Jordan: A cross-sectional study
Source: PLoS One. 2020 Mar 18;15(3):e0230421. doi: 10.1371/journal.pone.0230421 (PMC7080244; doi:10.1371/journal.pone.0230421)
Supplement: S10 Table — (DOCX) [file pone.0230421.s010.docx]

S10 Table. Reasons for non-use of family planning (n=402)

|  | n | % |
| --- | --- | --- |
| In pregnancy | 100 | 24.9 |
| In postpartum period | 67 | 16.7 |
| Wants to more children | 128 | 31.8 |
| Fear of infertility | 32 | 8.0 |
| Not having sex/ Infrequent sex | 10 | 2.5 |
| **Fertility-related reason** |  |  |
| Menopausal/ Hysterctomy | 27 | 6.7 |
| Sub fecund/ infecund | 27 | 6.7 |
| <Opposition to use> |  |  |
| Respondent opposed | 7 | 1.7 |
| Husband opposed | 18 | 4.5 |
| Others opposed | 2 | 0.5 |
| Religious prohibition | 1 | 0.2 |
| **Side effects** |  |  |
| Interferes with body processes | 24 | 6.0 |
| Mood changes | 11 | 2.7 |
| Bloat & weight gain | 14 | 3.5 |
| Headaches, dizzy | 18 | 4.5 |
| Bleeding irregularity | 16 | 4.0 |
| Risk of cancer | 5 | 1.2 |
| Dangerous to health | 21 | 5.2 |
| Can't use because of pre-existing health issues | 12 | 3.0 |
| Fear of side effects | 8 | 2.0 |
| **Use related reasons** |  |  |
| Forget to take | 2 | 0.5 |
| Method failure risk | 0 | 0.0 |
| Difficult to use | 0 | 0.0 |
| Interferes with sexual experience | 1 | 0.2 |
| **Lack of Knowledge** |  |  |
| Knows no method | 2 | 0.5 |
| Knows no source | 0 | 0.0 |
| **Access reasons** |  |  |
| Lack of access/ too far / inconvenient to get | 1 | 0.2 |
| Too much time to obtain | 0 | 0.0 |
| Cost too much to use | 0 | 0.0 |
| **Provider/facility reasons** |  |  |
| Lack of female providers | 0 | 0.0 |
| Lack of privacy | 0 | 0.0 |
| Provider offers limited methods | 0 | 0.0 |
| Provider did not give method requested | 0 | 0.0 |
| Requested methods not available | 1 | 0.2 |
| Inadequate counseling | 0 | 0.0 |
| Negative experience with FP provider | 0 | 0.0 |
| **Other** |  |  |
| Other | 11 | 2.7 |
| Don't know | 0 | 0.0 |
