# Supplementary material for: Demand for family planning satisfied with modern methods and its associated factors among married women of reproductive age in rural Jordan: A cross-sectional study
Source: PLoS One. 2020 Mar 18;15(3):e0230421. doi: 10.1371/journal.pone.0230421 (PMC7080244; doi:10.1371/journal.pone.0230421)
Supplement: S11 Table — (DOCX) [file pone.0230421.s011.docx]

S11 Table. Perceived reasons for non-use of modern contraceptives in community (n=1,019)

|  | n | % |
| --- | --- | --- |
| Fear of health problems | 861 | 84.5 |
| What to more children to fulfill maternal role | 195 | 19.1 |
| Perception of self as fertile and desirable | 16 | 1.6 |
| Fear of being abandoned by the husband | 13 | 1.3 |
| Husband many take another wife | 46 | 4.5 |
| Other | 37 | 3.6 |
| Don’t know | 63 | 6.2 |
