# Supplementary material for: Demand for family planning satisfied with modern methods and its associated factors among married women of reproductive age in rural Jordan: A cross-sectional study
Source: PLoS One. 2020 Mar 18;15(3):e0230421. doi: 10.1371/journal.pone.0230421 (PMC7080244; doi:10.1371/journal.pone.0230421)
Supplement: S12 Table — (DOCX) [file pone.0230421.s012.docx]

S12 Table. Places for obtaining contraceptives among women currently using family planning (n=617)

|  | n | % |
| --- | --- | --- |
| **Public** |  |  |
| Gov. hospital | 17 | 2.8 |
| University hospital | 1 | 0.2 |
| CHC / PHC | 192 | 31.1 |
| Village health centre | 4 | 0.6 |
| Royal medical services | 8 | 1.3 |
| Other public | 0 | 0.0 |
| **Private** |  |  |
| Private Hospital | 5 | 0.8 |
| Private clinic | 70 | 11.3 |
| JAFPP | 67 | 10.9 |
| UNRWA clinic | 14 | 2.3 |
| Other non-governmental organization | 0 | 0.0 |
| Pharmacy | 38 | 6.2 |
| Other Private | 1 | 0.2 |

Note: CHC: Comprehensive health centre, PHC: Primary health centre
