# Supplementary material for: Demand for family planning satisfied with modern methods and its associated factors among married women of reproductive age in rural Jordan: A cross-sectional study
Source: PLoS One. 2020 Mar 18;15(3):e0230421. doi: 10.1371/journal.pone.0230421 (PMC7080244; doi:10.1371/journal.pone.0230421)
Supplement: Attachment — Submitted filename: Response to Editors_Dec11.docx [file pone.0230421.s013.docx]

**Response to Editors (11/12/2019)**

PONE-D-19-25354R1

Demand for family planning satisfied with modern methods and its associated factors among married women of reproductive age in rural Jordan: A cross-sectional study Ms. Makiko Komasawa

| Comments | Response |
| --- | --- |
| 1) We note that you have included affiliation symbols '¶' and '‡', however no affiliations have been linked to them. Please amend these symbols to link an affiliation to them or remove if added in error. | It was added in error. We removed them. |
| 2) Thank you for providing the following data availability statement:  "No - some restrictions will apply  The dataset used in this manuscript is available upon request, with ethical restriction. To request data, contact Professor Motoyuki Yuasa, Department of Public Health, Faculty of Medicine, Juntendo University ([moyuasa@juntendo.ac.jp](mailto:moyuasa@juntendo.ac.jp))."  Before we proceed, please address the following points: |  |
| a) Please kindly clarify in detail the reasons for data restriction (e.g., data contain potentially sensitive information, etc.) and who has imposed them (e.g., an ethics committee).  b) Please also provide a non-author point of contact (e.g., data access committee, ethics committee, or other institutional body) where data request may be made. Note that it is not acceptable for the authors to be the sole named individuals responsible for ensuring data access. | We revised the part as follows:  .  “Data cannot be shared publicly because of containing potentially sensitive information by the decision of the ethics committee of Juntendo University. Data are available from Prof. Myo Nyein Aung ([myo@juntendo.ac.jp](mailto:myo@juntendo.ac.jp)v), Global Health Promotion Research Center, Faculty of International Liberal Arts, Juntendo University, for researchers who meet the criteria for access to confidential data.” |
