# Supplementary material for: Demand for family planning satisfied with modern methods and its associated factors among married women of reproductive age in rural Jordan: A cross-sectional study
Source: PLoS One. 2020 Mar 18;15(3):e0230421. doi: 10.1371/journal.pone.0230421 (PMC7080244; doi:10.1371/journal.pone.0230421)
Supplement: Attachment — Submitted filename: Response to Reviewers_Jan25.docx [file pone.0230421.s014.docx]

**Response to Reviewers**

PONE-D-19-25354

Demand for family planning satisfied with modern methods and its associated factors among married women of reproductive age in rural Jordan: A cross-sectional study

Makiko Komasawa, MUP, Ph.D; Motoyuki Yuasa, M.D., Ph.D.; Yoshihisa Shirayama, Ph.D.; Miho Sato, MA, MPH, Ph.D.; Yutaka Komasawa; Malak Alouri, M.D.

Reviewers’ comments and responses

| Reviewer 1 | |  |
| --- | --- | --- |
| No | Reviewer’s Comments | Response |
| 1 | This revised manuscript has largely addressed prior critiques but would still benefit from some final polishing and clarification before acceptance. The manuscript also need further review by a native English speaker as there are several areas with subject-verb disagreement and other grammatical errors or awkward phrasing. | Thank you for your second review.  The second revised version was checked by the a native English speaker who has the reproductive health background. |
| 2 | Abstract: |  |
|  | As an example of awkward phrasing and need for editing, the sentence, “A filed survey, through two-stage stratified sampling of village and household levels and systematic random sampling for households, was conducted from September to October 2016.” would benefit from being revised to “Two-stage stratified sampling with random sampling at household stage was used for this field survey conducted between September and October 2016.” | We received the English native speaker’s check for the entire manuscript.  Thank you for your providing model rephrasing. We utilized it as follows:.  “A two-stage stratified sampling with random sampling at the household stage was used for this field survey which was conducted between September and October 2016.” |
|  | In the Results, the statement “In addition, local women were aware of and easily access public health centres in their areas.” needs proportions to put the data into perspective for this sample. | We revised as follows:  “In addition, 95.3% of local women were aware of the presence of health centres that were mostly located in a 10-minute walking distance.” |
| 3 | Introduction: |  |
|  | Though the authors mention that a professional editor reviewed this revised manuscript, there are still multiple misspellings, issues with subject-verb agreement, and awkward phrasing (example: “” | We asked the native English speaker to review the whole manuscript. |
|  | The statement, “Generally, low education level and low economic status cause lower mDFPS coverage” needs to be revised and referenced as low education and economic status are not causal factors of low modern FP use but are correlates. | We revised it as follows:  “He estimated that mDFPS accounted for 47% of women worldwide and illustrated that in general higher mDFPS coverages were negatively associated with education and wealth.” |
| 4 | Methods: |  |
|  | This section has markedly improved. However, please capitalize proper nouns like Irbid Governorate and discuss the setting in present verb tense (current phrasing states “Irbid governorate was located in northern Jordan” – isn’t Irbid Governorate still located in northern Jordan?). | We received the native check here. Please refer the revised manuscript. |
|  | Please add detail to specify whether households were mapped to enable random selection and what was done if a selected household did not contain an eligible woman for participation. While an earlier manuscript is referenced, it would be helpful to readers here to be able to see in the text that this is truly a probabilistic sample. | In new Line 115, we revised as follows:  “The second stage involved selecting households by systematic random sampling in each village, using the household frame of the 2015 Jordan Census which was the same as the methodology used in the JPFHS 2012 [8].”  In new Line 120, we added the following phrase,  “When a household did not contain a woman eligible for participation, the household was replaced by the nearest one.” |
| 5 | Results: |  |
|  | No comments. | Thank you for your confirmation. |
| 6 | Discussion: |  |
|  | The Limitations section still needs attention. The dependent variable, whether a woman was using a method, relies on participant report and this needs to be acknowledged as a limitation. | In new Line 369, we revised as follows:  “The most significant limitation of this study may be possibility of containing biases from self-reporting by local women, such as practicing either modern or traditional contraception, fertility preferences, or the wantedness of recent births/pregnancies. Some bias may have been included; therefore, mDFPs may have been underestimated [35].” |

| Reviewer 2 | |  |
| --- | --- | --- |
| No. | Reviewer’s Comments | Response |
| 1 | This is the second time that I review this manuscript. I feel the manuscript improved greatly. My comment is attended. No further comments. | Thank you for your approval. |

From us

| No | Comments | Response |
| --- | --- | --- |
| 1 | Table 1 | We changed the format of Table 1  We corrected the figures related to “Monthly household income” |
| 2 | Table 2 | We corrected the figures related to “Age” and “Monthly household income” in Table 2. |
| 3 | Table 3 | We added “Missing” for Number of living children. |
