# Supplementary material for: Demand for family planning satisfied with modern methods and its associated factors among married women of reproductive age in rural Jordan: A cross-sectional study
Source: PLoS One. 2020 Mar 18;15(3):e0230421. doi: 10.1371/journal.pone.0230421 (PMC7080244; doi:10.1371/journal.pone.0230421)
Supplement: Attachment — Submitted filename: Response to Reviewers_Feb2.docx [file pone.0230421.s015.docx]

**Response to Reviewers**

PONE-D-19-25354

Demand for family planning satisfied with modern methods and its associated factors among married women of reproductive age in rural Jordan: A cross-sectional study

Makiko Komasawa, MUP, Ph.D; Motoyuki Yuasa, M.D., Ph.D.; Yoshihisa Shirayama, Ph.D.; Miho Sato, MA, MPH, Ph.D.; Yutaka Komasawa; Malak Alouri, M.D.

Reviewers’ comments and responses

| Reviewer 1 | |  |
| --- | --- | --- |
| No | Reviewer’s Comments | Response |
| 1 | The authors have responded to all comments. However, the response may have been done in haste as there are still seeming inaccuracies that should be remedied by careful review.  For example, the correction regarding Westoff et al.'s findings regarding modern contraceptive method prevalence and "that in general mDFPS is negatively associated with wealth and education." Shouldn't this be positively associated as women from higher wealth quintiles and with higher education levels are MORE likely to use modern FP methods? | Thank you for your careful comment  We reviewed the manuscript and received check by the native English speaker and revised the sentence as follow, by keeping the word “mDFPS.”  “He estimated that mDFPS accounted for 47% of women worldwide and illustrated that in general lower education level and lower economic status leads to lower mDFPS coverage.” |
