# Supplementary material for: Demand for family planning satisfied with modern methods and its associated factors among married women of reproductive age in rural Jordan: A cross-sectional study
Source: PLoS One. 2020 Mar 18;15(3):e0230421. doi: 10.1371/journal.pone.0230421 (PMC7080244; doi:10.1371/journal.pone.0230421)
Supplement: Attachment — Submitted filename: Response to Reviewers_Feb22.docx [file pone.0230421.s016.docx]

**Response to Reviewers**

PONE-D-19-25354

Demand for family planning satisfied with modern methods and its associated factors among married women of reproductive age in rural Jordan: A cross-sectional study

Makiko Komasawa, MUP, Ph.D; Motoyuki Yuasa, M.D., Ph.D.; Yoshihisa Shirayama, Ph.D.; Miho Sato, MA, MPH, Ph.D.; Yutaka Komasawa; Malak Alouri, M.D.

Editor comments

| No | Comments | Response |
| --- | --- | --- |
| 1 | There has been a marked improvement and is eaier to read and understand.  However between the orginal version and R1 there has been a change of p value in Table 2 for age: previously reported as 0.012 and now reported as 0.233. However, the narrative is still saying 'Two variables (i.e. age and duration of current residence) showed statistically significant differences between the two groups, whereas years of schooling, work experience, and household income did not.  Please reconfirm the p value for age or adjust the narrative. | We very much appreciated your careful reviews and suggestions.  We double-checked it and the p-value (0.233) for age is correct.  Therefore we adjusted the texts in line 204-205 as follows:  “One variable (i.e. ‘duration of current residence’) showed statistically significant difference between the two groups; whereas, age, education level, work experience, and household income did not.” |
| 2 | Spacing in Tables 3, and 6 after the symbol for less than or more than should be deleted. | We deleted the unnecessary spaces and additionally changed symbol ‘≤’ and ‘≥’ from Japanese version to English version. Please kindly check Table 3 and 6 in the revised manuscript. |
| 3 | Please harmonize decimal places for were then 1.8 times compared to (1.60 times) | We changed the both figures to ‘1.83’ and ‘1.59’. |
